# Notes and Notices

**Published:** 1894-11

**Authors:** 


### NOTES AND NOTICES.

Dr. Milton Powel announces his removal from 251 to 163 West Seventy-sixth Street, New York.

To the Medical Profession.—The controversy precipitated upon me by Dr. H. F. Biggar brings out an echo of the much and widely discussed problem of the "dispensary evil." I indorse, and as dispensary physician act upon, the theory that none are proper dispensary patients to be treated free except the worthy poor; that to extend its charitable help to the selfish and grasping, who can afford to pay, is a wrong to charitable donors and to practicing physicians, as well as a demoralizing influence upon society.

Dr. Biggar indorses and acts upon the theory that the proper use and sole object of a free dispensary is to gather in subjects for operation by the specialists as clinicians, and has, as you know, preferred charges against me because I, as dispensary physician, have refused at his demand to receive and treat those who can afford to pay, while I have opened the door wide to all who need treatment and cannot pay.

I believe that the institution he represents has no right to use the revenues secured by it from the Huntington bequest and other benevolent donors in aid of the worthy poor, in free service to the rich as an inducement to such to submit themselves to vivisection to the end of building the college up at the expense of its rivals. To get a concensus of the unprejudiced judgment of the profession at large, I address this card to the leading and reputable practitioners of the city, regardless of distinction as to "*pathy*," and ask an expression of opinion upon the questions presented. If you will kindly favor me with a statement of your views, I will not only be made grateful, but you will contribute to the proper settlement of the important issue presented. Your early reply is solicited. Very respectfully,

H. D. Bishop, M. D., 89 Euclid Avenue.

Cleveland, O., October 19th, 1894.

Having his vermiform appendix removed has been rather a good thing for Oscar Tully, of Yardville, N. J., for the obstruction was found to be a large pearl, which he must have swallowed in an oyster, and for which he has refused two hundred dollars.

Dr. Clarke begs to announce that he has taken consulting rooms in the city at 3 Newman's Court, 73 Cornhill, E. C., where he will attend on *Tuesdays* and *Thursdays*, between the hours of 11 and 3, commencing on Tuesday, October 16th.

On *Mondays, Wednesdays, Fridays,* and *Saturdays* Dr. Clarke will attend during the same hours (11 to 3) at his residence, 30 Clarges Street, Piccadilly, W.

LONDON, October, 1894.

HYDROGEN DIOXIDE. $H_2 O_2$. By L. D. Kastenbine, A. M., M. D., Professor Chemistry, Urinology, and Medical Jurisprudence Louisville Medical College; Professor Chemistry Louisville College Pharmacy. Published by *Louisville Medical Monthly*, July, 1894.

This remarkable liquid, which contains the greatest percentage of oxygen of any compound known, was, for some time, considered as a mere solution of oxygen in water, and consequently was called oxygenated water. It was afterward obtained free from water, and found to be a definite chemical compound of hydrogen and oxygen, and differing from water in containing twice as much oxygen. In this state it is a heavy, oily liquid, readily decomposing at ordinary temperatures, but if heated, with explosive violence, being converted into ordinary water and oxygen gas. When poured into water it sinks, being nearly half again as heavy as that liquid, but is miscible in all proportions with it. It has a somewhat bitter, astringent taste, and is colorless, transparent, and without odor. It contains 94 per cent. of oxygen gas by weight, and will yield 475 times its volume of that gas. It bleaches the skin, hair, ivory, and destroys organic coloring matter, pus, and all organisms with which it comes in contact by liberating oxygen gas in a nascent or active state. It is resolved into oxygen and water by certain metals, such as gold, platinum, silver, and mercury in a state of fine subdivision, although the metals themselves undergo no change whatever. If the oxides of these same metals are brought into contact with it, not only does the hydrogen dioxide lose oxygen and become water, but the oxides lose their oxygen and are reduced to the metallic state, thereby evolving an additional amount of oxygen.

Strange as it may appear, with all its energetic oxidizing action it has no effect on phosphorus, a substance which is so readily oxidized by the air.

The preparations found in commerce are only solutions of this compound in water, and sold in different degrees of concentration or strength, rated by the number of volumes of oxygen gas they can be made to yield. A fifteen-volume solution is one that will give off fifteen volumes of gas from one volume of the solution. A ten-volume solution will yield ten pints of oxygen gas from one pint of the solution, and so on.

These solutions, although more stable than mere concentrated preparations, nevertheless decompose and lose their nascent oxygen on which its powerful antiseptic powers depend, and consequently we find the commercial brands varying considerably from their reputed strengths. *The solution I find containing the greatest percentage of available oxygen is the preparation known as Marchand's, which, when perfectly fresh, is about a fifteen-volume solution.*

There are quite a number of different methods of preparing aqueous solutions of this interesting compound besides the original method of Thenard, the discoverer. Usually, however, barium dioxide in the hydrated state and purified from all foreign matter is decomposed by such acids as will make an insoluble compound with it. The United States Pharmacopœia has adopted this compound under the official title of Aqua Hydrogenii Dioxodi, gives a process of preparing it, and describes it as a slightly acid aqueous solution of hydrogen dioxide, containing, when freshly made, about three per cent. by weight of the pure anhydrous dioxide, corresponding to about ten volumes of available oxygen. It is made by the action of phosphoric acid upon barium peroxide. It must be borne in mind that it is essential to employ a small amount of free acid to preserve these solutions, but if too large a quantity it would be a source of irritation when applied to denuded surfaces and inflamed mucous membranes, and consequently, officially, a preparation requiring more than 0.5 c. c. of volumetric caustic potash solution to neutralize .50 c. c. of it does not come up to the United States Pharmacopœia standard.

*Of the various brands of commercial dioxides I have examined, I find Marchand's to be the one which yields the largest amount of available oxygen under all conditions of exposure, and the one which contains the minimum percentage of free acid. All the marketable articles I have seen are free from barium compounds, but the majority do not come up to the fifteen-volume standard, but are six, eight, ten, and twelve-volume solutions.*

In addition to its medical uses, hydrogen dioxide can be employed to detect blood, in conjunction with freshly prepared tincture of guaiac. Although tincture of guaiac turns blue with a variety of substances, blood is not one of them. So in testing for a stain—say on clothing—moisten the spot with water, and afterward apply a piece of white filter paper; the slightest straw-colored stain on the paper suffices. Now add to the spot on the paper a few drops of the guaiac tincture—no coloration. Add a few drops of solution of peroxide, when instantly the spot turns of a deep azure blue. Of course, if the spot turns blue by the guaiac alone it cannot be due to blood, yet it is possible blood may be present with some other substance which has that property, and hence the employment of peroxide, in that case, would be a source of fallacy. If there is no bluing by guaiac and peroxide together, then absolutely no blood is present.

Hydrogen dioxide can be determined quantitatively by permanganate of potassium solution acidified by sulphuric acid, and the quantity of oxygen gas evolved measured in an instrument called a nitro-meter, and calculated for normal pressure and temperature. One-half the oxygen evolved comes from the dioxide and the other half from the permanganate solution.

Another method, and the one commonly employed, is to add a volumetric solution of permanganate of potassium from a burette to a measured portion of the hydrogen dioxide solution, diluted with water and acidulated with sulphuric acid, until the permanganate solution is rendered colorless, and then a few drops more of that re-agent employed till a permanent faint pink coloration is given to the dioxide solution to indicate the completion of process. A slight calculation will give the strength of solution. There are other methods, but the two indicated are the best.

A solution of peroxide of hydrogen is usually tested by pouring a drachm of it in a clean test tube, together with an equal quantity of ether, then pouring into the tube a few drops of bichromate of potassium solution, and shaking the tube, when the ethereal layer will become of a beautiful azure color, due to the formation of perchromic acid which dissolves in the ether.

To a few drops of nitrate of silver solution add aqua ammonia enough to precipitate the oxide of silver, then add hydrogen peroxide when finely divided metallic silver separates. A solution of titanic acid in oil of vitriol and diluted will yield a yellow color when added to solutions of the peroxide.

Dr. Wm. R. Powel has removed from Philadelphia to Palatka, Florida.

Dr. C. H. Oakes has removed from Clinton, Mass., to Livermore Falls, Maine.

Rev. Henry H. Beach, son-in-law of the venerable Dr. P. P. Wells, has removed from Golden, Col., to Charles City, Iowa.

Dr. E. E. Reininger has removed from 1093 Taylor Street to 353 South Oakley Avenue, Chicago, Illinois.

Dr. M. D. Satterlee has removed from Sharpsville, Penna., to 715 Georgia Avenue, Chattanooga, Tenn.

Dr. Thomas G. Roberts has removed from Washington, Iowa, to 99 Thirty-seventh Street, Chicago, Illinois.

---

### FUN FOR DOCTORS.

Was No Doctor.—A short time ago a young lady was troubled with a boil on her knee which grew so bad that she thought it necessary to call a physician. She had formed a dislike for the family physician, so her father suggested several others, and finally said that he would call in the physician with the homœopathic case who passed the house every day. They kept a sharp lookout for him, and when he came along he was called in. The young lady modestly showed him the disabled member. The little man looked at it and said:

"Why, that's pretty bad."

"Well," she said, "what must I do?"

"If I were you," he answered, "I would send for a physician. I am a piano tuner."—*Louisville Courier Journal.*

Friend—"Well, Doc, how's business?"
Doctor—"Fine. Got two new cases in the next room."
Friend—"What, small-pox?"
Doctor—"No, champagne."—*Truth.*

